# Experimental Investigation of Technological Indicators and Surface Roughness of Hastelloy C-22 after Electrical Discharge Machining Using POCO Graphite Electrodes

**DOI:** 10.3390/ma15165631

**Published:** 2022-08-16

**Authors:** Rafał Nowicki, Rafał Świercz, Dorota Oniszczuk-Świercz, Marek Rozenek

**Affiliations:** Institute of Manufacturing Technology, Warsaw University of Technology, 00-661 Warsaw, Poland

**Keywords:** electrical discharge machining, EDM, POCO graphite, surface roughness, material removal rate, tool wear rate

## Abstract

Modern industry is focused on looking for new and effective technologies to manufacture complex shapes from alloys based on nickel and chromium. One of the materials widely used in the chemical and aerospace industry is Hastelloy C-22. This material is difficult to machine by conventional methods, and in many cases, unconventional methods are used to manufacture it, such as electrical discharge machining (EDM). In the EDM process, the material is removed by electrical discharges between a workpiece and a tool electrode. The physical and mechanical properties of the tool electrodes have a direct impact on the process efficiency, machining accuracy, and surface roughness. Currently, there has been a significant increase in the use of graphite as a material for tool electrodes due to the low purchase cost of the raw material, good machinability, and high sublimation temperature. In this work, an experimental investigation of the influence of the grain size of the graphite tool electrode on material removal rate (MRR), tool wear rate (TWR), and surface roughness (*Ra*) of Hastelloy C-22 was carried out. Two POCO graphite tool electrodes with a grain size of 1 µm (AF-5) and 10 µm (S-180) were used. Based on the experimental studies, empirical models describing the influence of machining parameters on technological indicators and the condition of the surface texture were determined. The research indicates that graphite with a larger grain provides higher process efficiency with high relative wear of the tool electrode. The lowest surface roughness was obtained for graphite with a smaller grain size (AF-5). The analysis of the machining parameters proves that the discharge current and pulse duration are the main factors determining the MRR and *Ra* values for both AF-5 and S-180 graphite. The time interval is the dominant parameter with regard to the relative wear of the graphite electrode.

## 1. Introduction

Electrical discharge machining (EDM) is an unconventional process widely used in the manufacturing industry to accurately machine complex three-dimensional components with extremely hard materials, as long as they conduct electricity [1,2,3,4]. The EDM process is often used to manufacture parts that are difficult to machine by conventional methods, such as injection molds, dies, and aerospace and surgical components [5,6,7,8]. In the EDM process, material is removed from the workpiece through a series of electrical discharges occurring between two separate electrodes immersed in a dielectric medium and connected to a current pulse generator. One electrode is the workpiece and the other is the tool electrode. The electrical discharges that occur in the sparking gap cause local melting and evaporation in both the workpiece and the material of the tool electrode. The result is a mapping of the shape of the tool electrode on the workpiece. The surface texture after electrical discharge treatment is characterized by overlapping spherical craters from single discharges. The volume of material removed from the craters depends on the EDM parameters, such as discharge current *I*, discharge voltage *U*_c_, pulse duration *t*_on_, and time interval *t*_off_; the tool electrode material; and the type of dielectric [9,10,11,12,13,14].

The mechanical and thermo-physical properties of the tool electrode material have a considerable influence on the EDM process performance in terms of material removal rate, tool electrode wear, and surface roughness of the workpiece [15,16,17,18,19]. The main properties of the electrode material taken into account are electrical and thermal conductivity, melting and boiling temperature, density of the material, and thermal expansion. Higher thermal conductivity reduces the temperature generated on the surface of the tool electrode. Thus, materials with higher thermal conductivity and density are preferred for tool electrodes, because they achieve lower electrode wear. Due to the high temperatures on the electrode surface during electric breakdown, the material should have a high melting point and low thermal expansion, which will reduce wear and ensure accurate shape mapping [20]. The tool electrode should also have good electrical properties. Higher electrical conductivity has a positive effect on reducing the tool wear and increasing the material removal rate. Another important factor in choosing the proper electrode material is its machinability. The cost of an element manufactured by the EDM process strictly depends on the cost of the tool electrode, which includes the cost of the raw material and tool production. Materials that are difficult to machine or expensive are not suitable for tool electrodes [21]. The electrodes used in EDM applications are generally made of copper or graphite, because they have good electrical and thermal conductivity and a high melting temperature [8]. The share of graphite electrodes in the EDM process is steadily increasing due to the low purchase cost of the raw material, good machinability, and high sublimation temperature. In the USA, approximately 95% of sinker EDM applications use graphite as an electrode material [22]. Haron [23] compared copper and graphite electrodes during EDM of XW42 tool steel. He reported that the graphite electrode was suitable for finishing operations due to its lower surface roughness. The electrode tool wear and material removal rate are greater for copper electrodes compared to graphite.

Graphite electrodes are classified into grades. The size of the graphite grain significantly affects the surface integrity, tool wear rate, and material removal efficiency. Torres [24] studied the influence of graphite tool polarity on the surface integrity of Inconel 600 and material removal efficiency. The results revealed that negative polarity led to a higher material removal rate, and it was recommended to use positive polarity when a low tool wear rate and good surface finish are desired. Aas [25] compared two graphite electrodes with different grain sizes for machining turbine vanes made of Ni alloy. It was reported that a graphite electrode with finer particles provided a significantly lower tool wear rate, but also lower material removal efficiency. Amorim [26] investigated the influence of electrical parameters and negative polarity in EDM of Ti6Al4V alloy using special graphite electrodes with particle sizes of 3, 10, and 15 µm. The best results for material removal efficiency, surface roughness, and tool wear were obtained with the 10 µm particle size graphite electrode. The researchers explained that increasing the size of the grain from 3 to 10 µm promotes efficient machining by the detachment of graphite particles from the electrode after the end of the electric discharge, as a result of the spalling phenomenon. The particles then collide with the molten pool on the workpiece and assist in the ejection of molten material from the crater. Increasing the grain size from 10 to 15 µm causes excessive contamination of the sparking gap with graphite grains, which causes short-circuit pulses.

This paper focuses on analyzing the influence of the grain size of POCO graphite electrodes on the technological indicators and the condition of the surface texture of Hastelloy C-22 after electrical discharge.

Hastelloy C-22 is a nickel–chromium alloy that is difficult to machine by conventional methods of treatment due to its high melting point and hardness. For this reason, EDM is frequently used to machining such materials. The selection of this material was made by considering its industrial importance in chemical and aerospace industries [12]. Current scientific research has not taken up the topic of implementing EDM technology for machining of Hastelloy C-22 with POCO graphite electrodes. For this reason, the undertaken research topic has an innovative character. The research results could make a significant contribution to the development of knowledge on erosion machining of nickel- and chromium-based superalloys with POCO graphite electrodes and be adapted in modern erosion centers.

## 2. Materials and Method

The purpose of the experimental research was to determine the influence of the electrical parameters and grain size of POCO graphite electrodes on the material removal rate (MRR), tool wear rate (TWR), and surface roughness (*Ra*) of Hastelloy C-22 after electrical discharge machining. Hastelloy C-22 is a nickel-based alloy widely used in the chemical and aerospace industries. The alloy is characterized by excellent resistance to pitting and oxidation, and crevice and stress corrosion [27,28]. The chemical composition of Hastelloy C-22 is presented in Table 1 [29].

**Figure 1:**
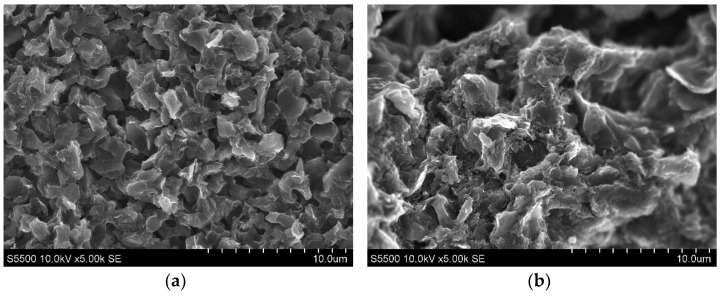
Graphite microstructure at ×5000 magnification for the samples: (**a**) AF-5; (**b**) S-180.

Experimental investigations were performed using a Charmilles Form 2LC ZNC machine. Manufactured samples made of Hastelloy C-22 had dimensions of Ø 10 × 2 mm. Two POCO graphite tool electrodes with grain size of 1 µm (AF-5) and 10 µm (S-180) were used in the experimental research. Figure 1 shows the photos taken on the Hitachi SU 3500 scanning microscope showing the differences in the grain microstructure of graphite electrodes, which were used in the experimental research. The main physical properties of the electrodes are presented in Table 2. The tool electrodes had a rectangular section of 10 × 10 × 15 mm. The samples and electrodes were lapped and polished before each experiment. A commercial EDM fluid, 108 MP-SE 60, was used as a dielectric.

### Machining Parameters

**Figure 2:**
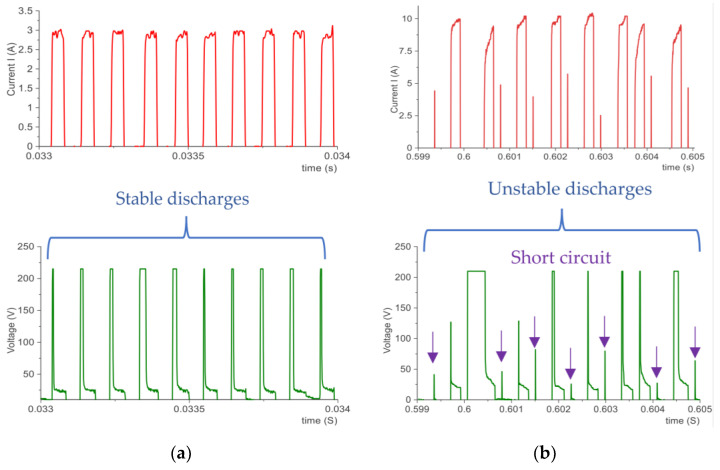
Current and voltage waveforms recorded during EDM for (**a**) stable and (**b**) unstable machining parameters.

A preliminary manufacturing test was conducted to find a stable range of machining parameters to be used as part of the experiment. For this purpose, a measurement circuit was used to monitor the real values of discharge current *I*, discharge voltage *U*_c_, pulse duration *t*_on_, and time interval *t*_off_. Graphs of voltage and current waveforms for stable and unstable machining parameters are shown in Figure 2. The discharge current has a direct impact on the volume of the material removed. Based on our own research and a literature analysis, high current values are used for roughing operations in order to ensure optimal process efficiency with acceptable high surface roughness. The pulse duration is responsible for the amount of thermal energy delivered to the workpiece. Extending the pulse duration results in induction of more heat into the material, which, for the same current value, results in an increased diameter of the generated crater. The time interval is responsible for stabilizing the conditions in the sparking gap and deionizing the inter-electrode plasma channel.

Based on the preliminary tests, it was found that in the case of graphite electrodes, too long of a pulse duration and a short time interval destabilize the erosion process. This is directly related to the high contamination of the sparking gap with machining products and graphite grains, which leads to short-circuit pulses. A long time interval favorably affects the process stability. A short time interval prevents effective cleaning of machining products and detached graphite grains, which can remain in the gap between the electrodes, reducing the resistance of the dielectric medium and increasing the probability of short-circuit pulses. An important factor influencing the EDM process is the polarity of the electrode. With negative polarity, high rates of material removal and tool wear of the graphite electrode were observed. The polarity also directly influences the increase in surface roughness after machining. For the purposes of this research, it was decided to use positive polarity, which ensures lower surface roughness with graphite electrodes compared to negative polarity. The analysis of voltage and current waveforms in the preliminary tests allowed us to establish a stable range of machining parameters in the EDM process for finishing and semi-finishing machining of Hastelloy C-22 using POCO graphite electrodes. Table 3 lists the machining conditions of the setup.

Experimental studies on the influence of discharge current *I*, pulse duration *t*_on_, and time interval *t*_off_ on MRR, TWR, and *Ra* of Hastelloy C-22 after electrical discharge machining were conducted using Hartley’s experimental design, which has five levels and three input parameters. Table 4 shows the levels of machining parameters used in the experiment.

The arithmetic mean deviation of roughness profile *Ra* was measured for each machined surface. The measurement was performed on a Taylor-Hobson FORM TALYSURF Series 2 scan profilometer. The surface roughness on the sampling line of 12.8 mm was measured three times, and the arithmetic mean of the measured *Ra* values was determined.

**Figure 3:**
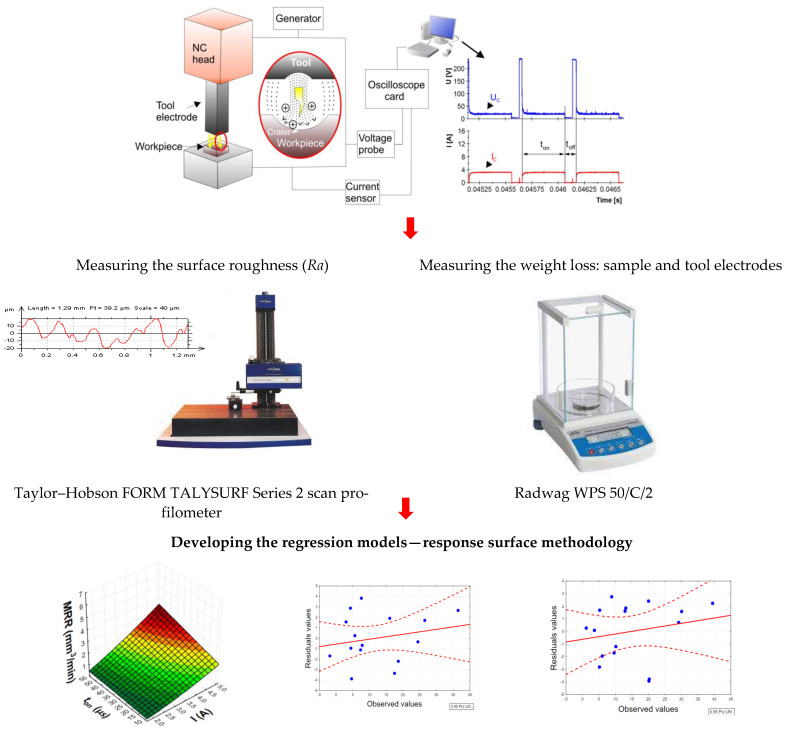
Schematic diagram of the experimental set-up.

The material removal rate (MRR) and relative tool wear (TWR) were determined based on the weight loss of the sample and the graphite electrode. The weight of the sample before and after the experimental test was measured three times, and the mean value was determined. Weight loss was measured on a Radwag WPS 50/C/2 electronic scale. Schematic diagram of the experimental set-up is presented in Figure 3.

The MRR value was determined based on the volume of material removed from the workpiece divided by the machining time (1):

(1) $$MRR=\frac{m_{1PO}-m_{2PO}}{\rho_{PO}\cdot \Delta t}\;\left(\frac{mm^{3}}{min}\right)$$

where *m*_1PO_ is the mass of the sample before machining; *m*_2PO_ is the mass of the sample after machining; *ρ*_PO_ is the density of the workpiece; and Δ*t* is the time to machine a sample to a depth of 0.5 mm.

The relative wear of the tool electrode was defined as the ratio of the volume of material removed from the electrode *Q*_ER_ and the workpiece *Q*_PO_ (2):

(2) $$TWR=\frac{Q_{ER}}{Q_{PO}}\cdot 100\;=\frac{(m_{1ER}-m_{2ER})\cdot \rho_{PO}}{(m_{1PO}-m_{2PO})\cdot \rho_{ER}}\cdot 100\;\left(\%\right)$$

where *m*_1ER_ is the mass of the tool electrode before machining; *m*_2ER_ is the mass of the tool electrode after machining; and *ρ*_ER_ is the density of the tool electrode.

## 3. Results and Discussion

The focus of these experimental studies was to determine the influence of the grain size of graphite electrodes and machining parameters on the technological indicators (MRR and TWR) and surface roughness (*Ra*) of Hastelloy C-22 after EDM. The research was divided into a few stages. In the first stage, we analyzed the material removal efficiency (MRR) and relative tool wear (TWR) of POCO AF-5 and POCO S-180 graphite electrodes. In the next stage, we determined the influence of the tested input parameters on the basic parameter describing surface roughness, *Ra*. Finally, we used response surface methodology to develop a mathematical model. A second-degree polynomial function was determined, describing the influence of the analyzed machining parameters on the selected output parameters. The results of the obtained research are presented and discussed in the following subsections.

### 3.1. Material Removal and Tool Wear Rates

The analysis of the experimental results shows that the material removal efficiency of the EDM process and the relative wear of the graphite tool electrodes varied in a wide range, strictly depending on the energy parameters and electrode grain size. The volume efficiency of the material removal rate for the AF-5 electrode was within the range of MRR = 0.26–3.81 mm^3^/min, while for the S-180 electrode, it was 0.26–4.21 mm^3^/min. In both cases, discharge current *I* was the most influential factor on MRR. The results of the experiments are presented in Table 5.

The POCO graphite electrode with a grain size of 10 µm provided a higher material removal efficiency than the graphite electrode with a grain size of 1 µm. With the same machining parameters, the S-180 electrode, compared to the AF-5 graphite, provided an average increase in process efficiency from 2 up to 13%. Due to the high sublimation temperature and low apparent density of graphite, it tended to lose particles from the electrode during the electro-erosion process. This was especially noticeable for graphite with large grain sizes. The detached grains that got into the gap were concentrated in the area with the highest electric field strength between the electrode and the workpiece. These particles could create voltage bridges and initiate an electrical breakdown. Frequent detachment of S-180 graphite grains could lead to more intense electric discharges and increased MRR. Moderate detachment of graphite grains during electrical discharges could also cause them to hit the pool of liquid material in the crater and more effectively remove the molten material. Similar dependencies were also observed by Amorim [26]. The larger grain size of S-180 graphite provided more impact energy to the pool of liquid material than did the AF-5 graphite, resulting in increased melt removal efficiency.

### 3.2. Analysis of Surface Integrity

**Figure 4:**
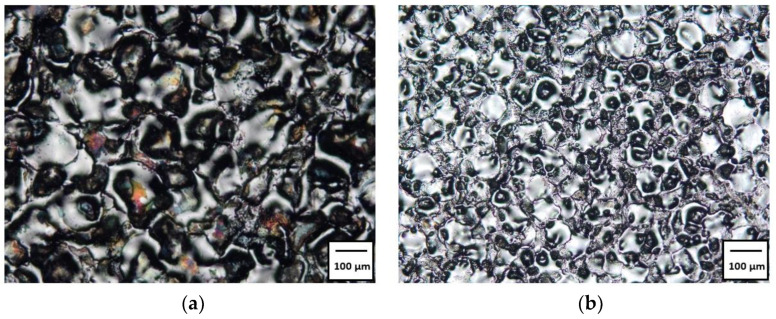
Surface texture of Hastelloy C-22 after EDM with S-180 (**a**) and AF-5 (**b**) graphite electrode for parameters: (**a**) *I* = 5 A, *t*_on_ = 30 µs, *t*_off_ = 37 µs; (**b**) *I* = 1.7 A, *t*_on_ = 30 µs, *t*_off_ = 37 µs.

In EDM machining, the material is melted and removed as a result of heat generated by the plasma channel during an electrical discharge. The superimposition of the traces of single electrical discharges creates an isotropic surface texture, consisting of a set of spherical craters (Figure 4). The condition of the surface layer has a significant impact on the operational and tribological properties.

**Figure 5:**
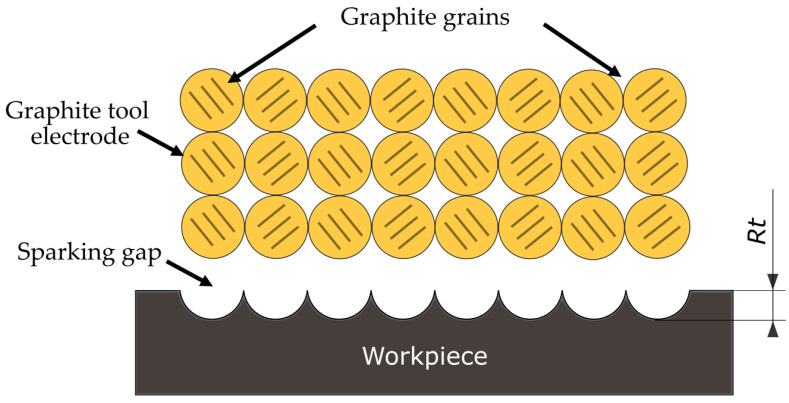
Illustration of mapping microstructure of graphite grains on surface of workpiece.

The research results showed a significant influence of the machining parameters and the grain size of graphite electrodes on the value of *Ra*. The *Ra* values for the AF-5 electrode were in the range of *Ra* = 1.44–3.52 µm. For S-180 graphite, the range was *Ra* = 2.07–4.21 µm. In all experiments, the *Ra* value obtained for the AF-5 electrode was lower than that for the S-180 graphite. The experiment was performed for the parameters corresponding to the conditions of semi-finished and finished EDM. With a low-power electric discharge, the graphite tool electrode is intended to reproduce the microstructure of the graphite grains on the machining surface (Figure 5). Graphite with a smaller grain and fewer pores has a lower surface roughness than graphite with a larger grain.

### 3.3. Statistical Models of MRR, TWR, and Ra

The research was carried out based on Hartley’s experimental methodology, which includes five levels and three input machining parameters. According to the adopted plan, 16 tests were carried out for each graphite electrode with different current intensity, pulse duration, and time interval values. In order to verify the repeatability of the process, two repetitions were carried out in the central part of the experiment.

Empirical models of a second-degree polynomial describing the influence of selected processing parameters on material removal rate and tool wear rate were designed. The degree of fit of the regression equation to the results of experimental tests is described by the correlation coefficient *R*. The value of *R* defines the statistical relationship between the input data of the tested process and the output data of the obtained regression equation. For each equation the correlation coefficient *R* was determined. As the value of *R* approaches unity, it better represents the variability of the study characteristic. The Fisher–Snedecor test (*F*) was used to examine the pertinence of the resulting correlation coefficient value. The value of function test *F* was compared with critical value *F*_kr_. *R* is significant when there is a relation *F*/*F*_kr_ ≥ 1 for the associated *p*-value (*p* = 0.05). The significance of individual terms of the equation was verified with Student’s t-test. The value of the function test *t* was determined and compared with the critical value of *t*_kr_ for the associated *p*-value (*p* = 0.05). The terms of the equation were significant when there was the relation *t* ≥ *t*_kr_ [30,31,32,33].

Response surface methodology was used to build regression models of the process. The equations are characterized by a high ratio of correlation *R* and very certain dependency. The ratio *F*/*F*_kr_ is much greater than one. This proves that the developed regression function has a good fit with the obtained test results. Table 6 summarizes the regression statistics for the designated equations.

**Figure 6:**
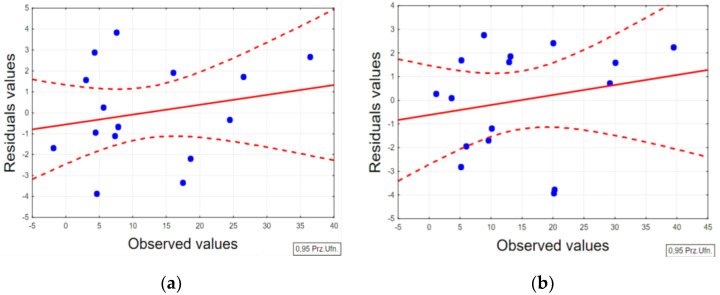
Plots of residual values as a function of observed values for TWR models of (**a**) AF-5 and (**b**) S-180 graphite electrodes.

For each determined equation, an analysis of the residual values was performed. Figure 6 shows example plots of residual values as a function of observed values for TWR models. For the determined statistical models, a small dispersion between the obtained test results and the observed values in the area of the adopted confidence level (95%) was observed.

**Figure 7:**
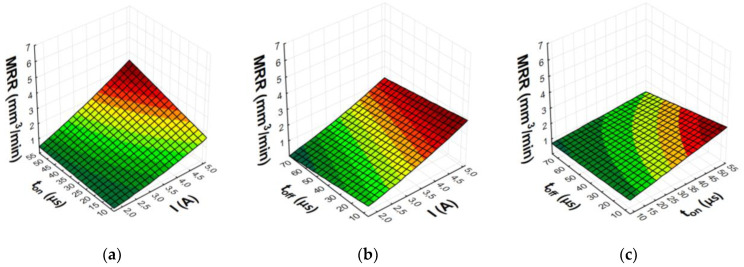
Graphical representation of regression equation for MRR of AF-5 graphite electrode: (**a**) *t*_off_ = 40.5 µs; (**b**) *t*_on_ = 31.5 µs; (**c**) *I* = 3.4 A.

**Figure 8:**
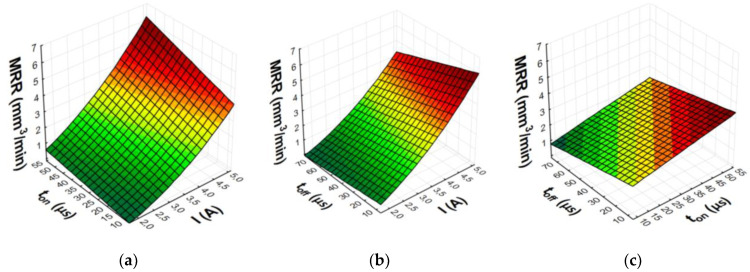
Graphical representation of regression equation for MRR of S-180 graphite electrode: (**a**) *t*_off_ = 40.5 µs; (**b**) *t*_on_ = 31.5 µs; (**c**) *I* = 3.4 A.

**Figure 9:**
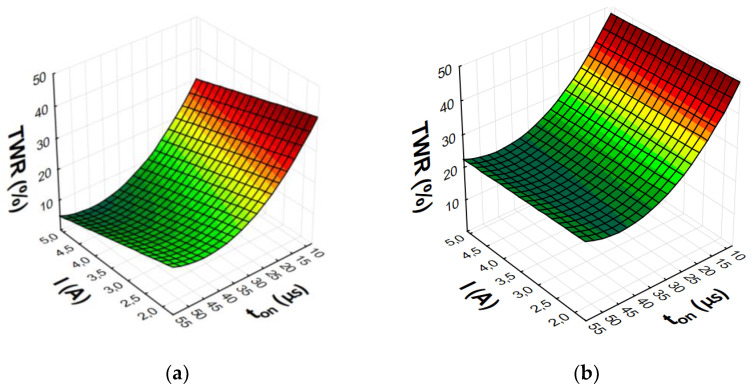
Graphical representation of regression equation for TWR of (**a**) AF-5 and (**b**) S-180.

**Figure 10:**
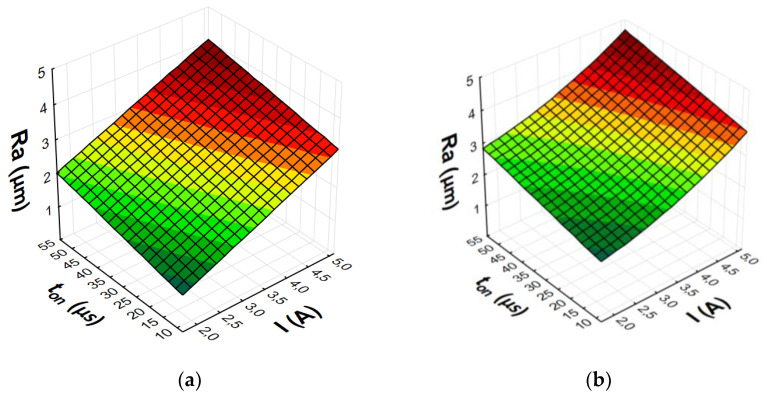
Graphical representation of regression equation for *Ra* of (**a**) AF-5 and (**b**) S-180.

After eliminating irrelevant terms of the equation, the final second-degree polynomial function was established to describe the influence of machining parameters on MRR, TWR, and *Ra* (3)–(8). Graphical representations of the calculated regression models are shown in Figure 7, Figure 8, Figure 9 and Figure 10.

Regression equations for the AF-5 graphite electrode:

*MRR* = 0.03 + 0.05 · *I*^2^ + 0.01 · *I* · *t_on_* − 0.0003 · *t_on_* · *t_off_* (mm^3^/min) (3)

*TWR* = 59 − 3.1 · *I* − 1.8 · *t_on_* + 0.02 · *t_on_*^2^ (%) (4)

*Ra* = −0.1 + 0.6 · *I* + 0.02 · *t_on_* (µm) (5)

Regression equations for the S-180 graphite electrode:

*MRR* = −0.5 + 0.2 · *I^2^* + 0.009 · *I* · *t_on_* − 0.005 · *I* · *t_off_* (mm^3^/min) (6)

*TWR* = 63 − 0.4 · *I* − 1.8 · *t_on_* + 0.02 *· t_on_^2^* (%) (7)

*Ra* = 1.5 + 0.02 · *t_on_* + 0.08 · *I^2^* (µm) (8)

The analysis and graphical interpretation of the designated regression models indicated that current intensity *I* was the dominant factor affecting MRR and TWR for both AF-5 and S-180 electrodes.

The increase in the current intensity *I* and the pulse duration *t*_on_ increased the material removal efficiency and the surface roughness (*Ra*) after machining. Low tool wear rate was achieved with high values of the current intensity *I* and the long pulse duration *t*_on_.

**Figure 11:**
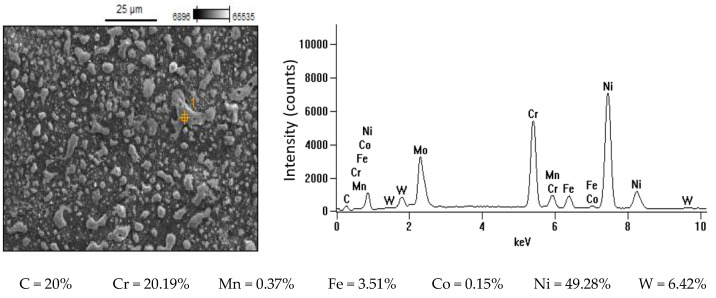
EDS spectrum of surface of AF-5 graphite electrode: *I* = 3.8 A, *t*_on_ = 55 µs, *t*_off_ = 37 µs.

Increasing the current intensity increased the diameter and power of the plasma channel, which generated a deeper melt and the removal of a larger volume of workpiece material. At low currents, a small amount of heat was generated, which was partially absorbed by the workpiece, and the rest was absorbed by the dielectric liquid. Pulse duration *t*_on_ was the second most important factor affecting machining efficiency. Increasing the pulse duration promoted efficient material removal by inducing more heat into the workpiece. Furthermore, the contribution of the time interval to electrode wear was greater than that of the discharge current. During machining, decomposition of the carbon dielectric by the thermal energy of discharge led to the deposition of carbon on the electrode surface, thus forming a protective layer that prevented excessive wear of the tool. A longer time interval favored a lower TWR due to carbon and workpiece material depositing on the electrode surface. The lowest wear value of the tool electrode was observed for experiment 12 and AF-5, in which the longest pulse duration, *t*_on_ = 55 µs, was set. The greater mass of the electrode after the experimental test was due to the deposition of carbon and melted workpiece material on the electrode surface. This was confirmed by the analysis of the EDS spectrum for sample 12, based on which the presence of the elements of the workpiece on the electrode surface was determined (mainly nickel, chromium, and molybdenum) (Figure 11). These elements can constitute an additional protective barrier preventing excessive wear of the tool electrode.

The value of *Ra* strictly depends on the energy of the electrical discharges. Increasing the current intensity causes deeper penetration of heat into the material and the formation of craters with larger diameters and depth. At low and high current values, significant changes in surface roughness were observed with increasing pulse duration. Increasing the time interval increased the diameter of the plasma channel and decreased the energy density. Craters created under such machining conditions were characterized by larger dimension and depth, which deteriorated the surface roughness.

## 4. Summary

The presented work was focused on determining the effect of graphite electrode grain size during EDM of Hastelloy C-22 using POCO graphite electrodes with different grain sizes (AF-5 and S-180). The influence of the grain size of graphite electrodes and machining parameters on MRR, TWR, and roughness parameter *Ra* was described and established. The material removal process and tool wear mechanism of graphite electrodes were analyzed. In the last stage of the research, second-degree polynomial functions were calculated to describe the influence of machining parameters on MRR, TWR, and *Ra*. Based on the analysis of the results obtained in the experimental research, the following conclusions were drawn:

1. Relative wear of the working electrode was greater for S-180 graphite due to the larger grain size, as well as lower resistivity and apparent density.
2. POCO AF-5 graphite electrode obtained the lowest surface roughness after EDM. With low electrical discharge energy, the graphite electrode mapped its grain microstructure on the machining surface. Graphite with a smaller grain and low relative porosity provided lower surface roughness.
3. The main determinants of MRR and *Ra* were discharge current *I* and, to a lesser extent, pulse duration *t*_on_. In the case of TWR, the most important parameters were pulse duration, followed by discharge current.
4. A long pulse duration reduced TWR due to the deposition of carbon and elements of the workpiece on the electrode surface, providing a protective layer to prevent excessive wear of the tool.
5. Increase of the discharge current *I* reduced the value of the relative tool wear for both the AF-5 and S-180 graphite electrodes.
6. An increase in the value of the current intensity *I* and the pulse duration *t*_on_ caused an increase in the roughness of the surface after EDM.
7. The developed predictive models for EDM of Hastelloy C-22 using graphite electrodes can be implemented for the construction of technological tables in modern erosion machines.

## Figures and Tables

**Table 1:** Chemical composition of Hastelloy C-22 (mass%) [29].

Ni	Cr	Mo	Fe	W	Co	Mn	C
56 Balance	20.0–22.5	12.5–14.5	2.0–6.0	2.5–3.5	2.5 max	0.5 max	0.015 max

**Table 2:** Physical properties of POCO graphite electrodes.

POCO Graphite	AF-5	S-180
Average grain size (µm)	1	10
Apparent density (g/cm^3^)	1.8	1.78
Electrical resistivity (µΩm)	21.6	13
Shore hardness	87	66
Flexural strength (MPa)	117	58

**Table 3:** Machining conditions.

Material electrode	POCO AF-5 (1 µm), POCO S-180 (10 µm)
Workpiece material	Hastelloy C-22
Discharge current *I* (A)	1.7–5
Pulse duration *t*_on_ (µm)	8–55
Time interval *t*_off_ (µm)	6–75
Discharge voltage *U*_c_ (V)	25
Open voltage *U*_0_ (V)	225
Tool polarity	Positive (+)
Machining depth *a*_p_ (mm)	0.5
Dielectric	EDM fluid 108 MP-SE

**Table 4:** Designed process parameters and their levels.

Level	EDM Parameter		
	Discharge Current *I* (A)	Pulse Duration *t*_on_ (µm)	Time Interval *t*_off_ (µm)
−1.68	1.7	8	6
−1	2.7	17	19
0	3.8	30	37
1	4	41	51
1.68	5	55	75

**Table 5:** The design of the experimental matrix with measurements: material removal rate (MRR), tool wear rate (TWR), and roughness *Ra*.

Ex. No.	EDM Parameters			Material of Electrode AF-5			Material of Electrode S-180		
	*I* (A)	*t*_on_ (µs)	*t*_off_ (µs)	MRR (mm^3^/min)	TWR (%)	*Ra* (µm)	MRR (mm^3^/min)	TWR (%)	*Ra* (µm)
1	2.7	17	19	0.74	26.48	1.9	0.84	30.04	2.53
2	2.7	17	51	0.57	24.44	2.03	0.64	29.16	2.37
3	2.7	41	19	1.13	4.43	2.21	1.19	5.12	2.6
4	2.7	41	51	0.89	5.63	2.52	0.92	5.99	3.24
5	4	17	19	1.46	18.62	2.38	2.82	20.26	2.57
6	4	17	51	1.64	17.46	3.06	1.84	20.11	2.97
7	4	41	19	3.10	2.99	3.42	3.40	3.62	3.43
8	4	41	51	2.59	4.29	3.07	2.82	5.21	3.32
9	1.7	30	37	0.26	16.03	1.44	0.27	20.03	2.07
10	5.0	30	37	3.81	7.57	3.52	4.21	8.82	4.21
11	3.8	8	37	0.86	36.44	2.35	0.99	39.46	2.94
12	3.8	55	37	2.87	−1.87	3.35	2.99	1.11	3.64
13	3.8	30	6	2.59	4.59	3.12	2.81	9.59	3.26
14	3.8	30	75	1.64	7.35	2.98	1.75	10.09	3.32
15	3.8	30	37	1.96	7.81	2.91	2.08	13.14	3.31
16	3.8	30	37	1.80	7.77	2.85	2.22	12.90	3.37

**Table 6:** Regression summary.

POCO Graphite	Investigated Parameters	Calculated Regression Statistics	
		Ratio *R*	*F*/*F*_kr_
AF-5	MRR	0.98	25.9
	TWR	0.98	25.0
	*Ra*	0.95	17.7
S-180	MRR	0.98	5.9
	TWR	0.98	15.7
	*Ra*	0.92	9.4
